# Comparative histopathology of climbing perch*, Anabas testudineus* challenged with *Aeromonas veronii* via IM and IP routes

**DOI:** 10.5455/javar.2025.l988

**Published:** 2025-12-25

**Authors:** Md. Ali Reza Faruk, Fatema Jahan, Maliha Farzana, K. M. Abdul Halim, Tamanna Tabassum, Tanvir Rahman, Salman Shahriar Nibir, Ishrat Zahan Anka

**Affiliations:** 1 Department of Aquaculture, Bangladesh Agricultural University, Mymensingh, Bangladesh; 2 Department of Aquaculture, Khulna Agricultural University, Khulna, Bangladesh; 3 Department of Fisheries, Ministry of Fisheries and Livestock, Dhaka, Bangladesh; 4 Department of Aquaculture, Chattogram Veterinary and Animal Sciences University, Chattogram, Bangladesh

**Keywords:** Histopathology, Intramuscular (IM), Intraperitoneal (IP), Clinical signs, Mortality

## Abstract

**Objective:**

The focal purpose of this investigation was to contrast the pathological changes from different histological observations of *Anabas testudineus* artificially infected with *Aeromonas veronii*.

**Materials and Methods:**

Intramuscular (IM) and intraperitoneal (IP) injection routes were used to challenge fish with three different bacterial concentrations, including 2.1 × 105, 2.1 × 105, and 2.1 × 104cfu ml^−1^ of bacteria to investigate the effects of various loads of *A. veronii* on the histopathological alterations in the skin-muscle, liver, and intestine of *A. testudineus* during 13 days of post challenge test. Two replicates (*n* = 10) were used for each of the IM and IP groups, corresponding to the three aforementioned bacterial loads, in the challenge test for this study.

**Results:**

The highest bacterial challenge (2.1× 104 cfu ml^−1^) groups from both IP and IM produced some prominent clinical signs, e.g., red spots, ulcers, and lesions on the body surface, and highest cumulative mortality (IP = 50% and IM = 40%) compared to the control groups having no pathological signs in all organs. Histopathological alterations observed under the light microscope revealed significant pathologies (e.g., vacuolation and necrosis) in all organs studied, particularly at the highest bacterial loads, compared to the other challenges and control groups. It suggests that varied bacterial loads can produce different types of pathology in various fish organs.

**Conclusion:**

*Aeromonas veronii* can cause mortality and remarkable pathological changes in different organs of *A*. *testudineus.* Findings from experimental infections can be used as an effective tool to predict the virulence of pathogens and to develop advanced prevention and health management strategies in aquaculture.

## Introduction

The total production of the world’s fisheries and aquaculture was recorded at 223.2 million tons in 2022, according to a recent report by [1]. There, 84% of the total inland production was obtained from aquaculture, with the majority (70%) contributed by the production of Asian countries. Among the major aquaculture-producing countries in Asia, Bangladesh has secured 2nd place for the world’s freshwater fish production (13.22 lakh tons, comprising 11.7% of the global total production). Bangladesh has also held the 5th position in a row for five times, although it has dropped (in Asia) from 3^rd^ to 5^th^ regarding the world’s total farmed aquatic animals’ harvesting [1,2]. Freshwater fish farming is a very common practice in Bangladesh. Among the many freshwater aquaculture finfish species in Bangladesh, the climbing perch (*Anabas testudineus*), also known as koi, has become the preferred and most profitable species for aquaculture farmers. It is due to this fish’s higher growth [3], comparatively short culture duration, high survival with its unique respiratory support [4], high market demand, and better nutritional value compared to native species [5]. Thus, this species has been contributing to the country’s overall increasing aquaculture demand.

A new variety of *A.* *testudineus*, commonly known as Thai koi, was introduced in Bangladesh from Thailand in 2002 and has opened a new scope for fish farmers, which was first reported in Mymensingh district, mostly through pond culture, and the production of this species was also found as profitable among farmers [6,7]. The production of *A*. *testudineus* was recorded at 46,457 Metric tons (MTs) in 2018 and 57,244 MT in 2022 [8,9], which clearly demonstrates the increasing farming trend of this species in pond aquaculture in Bangladesh. As a consequence of the rapid intensification of fish farming, subsequent risk factors have been faced by aqua farmers throughout the production process. Throughout the culture, *A*. *testudineus* can be subjected to various unfavorable challenges, which can lead to stress and disease outbreaks. Major disease incidence occurs in aquaculture due to the intensification and extension of farming practices [10], and as a result, disease can hinder the economic gain of many aquatic species at various scales [11]. In farmed koi in Bangladesh, diseases were reported for example, the disease called epizootic ulcerative syndrome (EUS), followed by some cases of tail and fin rot, and also some symptoms related to gill damage, skin erosion [12], and also the presence of some pathogenic bacteria [13] has been reported from both the indigenous and exotic *A. testudineus*.

Among different bacterial pathogens, the genus *Aeromonas*, from the Aeromonadaceae family, has garnered significant attention worldwide over the past few years due to its association with a high mortality rate in infected fish species [14]. Diverse *Aeromonas* spp. have been recognized for causing infection and diseases in fish [15] as a primary pathogen in different farmed fishes [16], and among many Aeromonads, *Aeromonas* *veronii* has been reported as a most virulent pathogen with a high percentage of mortality in fish (e.g., observed in Nile Tilapia) [17]. *Aeromonas* *veronii* was also detected as a significant pathogen from the aquaculture fish species (i.e., European seabass) [18] and for different freshwater ornamental fish species [19]. More recently, *A*. *veronii* infection in fish has been found to have an increased rate of infection, as well as very similar symptoms and histological observations, similar to those typically observed in *A. hydrophila* infections [20]. Several strains of *A*. *veronii* have been increasingly isolated from diseased fish (e.g., striped catfish [21], Largemouth bass [22]), and a range of diverse clinical signs and symptoms have been detected in infected fish. The common clinical signs for *A*. *veronii* infection have been detected as a form of ulcer, exophthalmia and hemorrhage, abdominal distention, and fin rot or tail rot in fish, whereas the symptoms are not always evenly shown with potential pathogenicity in different fish infected with diverse strains or isolates of *A*. *veronii* [20]. So, there is still scope for research considering the diversity of clinical signs and histological pathology in farmed fish infected with *A*. *veronii*. This type of information can be useful for better understanding the virulence of this pathogen and for comparing the state of histological lesions across infected fish. However, the information has not been explored adequately in aquaculture fish species in Bangladesh.

Recently, *A*. *veronii* has been isolated from diseased climbing perch* A*. *testudineus* in Bangladesh [23]. However, comparative histopathological studies using different challenge routes in *A*.* testudineus* from Bangladesh are scarce, and this type of comparative infection study in aquaculture species has yet to be explored in this context. This sector requires greater attention, not only for the health management aspects of this fish to prevent production losses due to bacterial infection, but also to ensure the welfare of the farmed fish species. Experimental infection can shed light on the mechanisms involved in the virulence and pathology of *A. veronii* in Thai koi, a susceptible host species. Considering this, the present study was set to compare visible pathological variations in different organs of Thai koi, *A*. *testudineus,* artificially infected with *A*. *veronii* via two different challenge routes.

## Materials and Methods

### Ethical approval

This study on climbing perch (*A. testudineus*) challenged with *A. veronii* was approved by the Ethics and Safety Review Committee (ESRC) of BAURES (Approval No. ESRC/57/FISH/2025).

### Bacterial preparation

Laboratory stock of *A*. *veronii* bacteria, previously isolated from diseased Thai koi have been tested in this study. The bacteria culture was done in Tryptic Soy Agar (TSA) agar media for 24 h at 25^°^^C^. Bacterial calculation was determined by following the serial dilution method and termed as colony forming unit (cfu ml^−1^). This cfu ml^−1^ unit was calculated from previously cultured bacterial suspension following the drop count method (Supplementary Information S1).

### Experimental infection

For this study, *A*. *testudineus* (average size of 85 ± 1.72 gm) were obtained from the healthy fish stocks from a commercial hatchery and farm named Sarnatala Hatchery located at Mymensingh. Duplicate groups of fish were deposited in 30 L tanks. Each tank was aerated, and fish were acclimatized for 4 days prior to being released into the tank. Throughout the experimental period, a commercially pelleted diet was provided to the fish at a rate of 2–3 times daily. To maintain good water quality, approximately one-third of the tank water was replaced daily. Dead fish were removed if observed, and siphoning was performed as needed to remove debris from the bottom of the tank.

Two routes (intramuscular, IM, and intraperitoneal, IP injections) were used in this experiment to perform the challenge test. The experiment was conducted following the ethical approval given by BAURES (ESRC/57/FISH/2025). The challenge was done by injecting the fish with three different concentrations of *A*. *veronii*, including 2.1 × 104, 2.1 × 105, and 2.1 × 104cfu ml^−1^. Two replicate fish groups (*n* = 10 each) were subjected to a challenge test via IM and IP routes by injecting the three bacterial loads mentioned above in this study (Table 1). A randomized distribution was followed to assign the fish individuals to their respective experimental study groups. Details of the experimental set-up for each challenge test group are mentioned in Table 1. However, no statistical test was performed in this study, given the comparatively small sample size (*n* = 10), which resulted in limited statistical power, and the nature of the data analysis.

**Table 1.** Details of experimentally infected and control fish groups.

Fish groups (Code)	Replicate	Challenged fish (number)	Challenge dose (Cfu ml ^−^^1^)/ PS
IM	1	10	2.1 × 104
	2	10	2.1 × 104
IM	1	10	2.1 × 105
	2	10	2.1 × 105
IM	1	10	2.1 × 106
	2	10	2.1 × 104
IP	1	10	2.1 × 104
	2	10	2.1 × 104
IP	1	10	2.1 × 105
	2	10	2.1 × 105
IP	1	10	2.1 × 106
	2	10	2.1 × 106
CF	1	10	PS
	2	10	PS

IM = Intramuscular injected fish, IP = Intraperitoneally injected fish, CF = Control Fish groups, and PS = Physiological Saline

The injection site for fish was selected as below the left dorsal fin, and injection was done, taking 0.1 ml of bacterial suspension (from low to high dose with a view to comparing the gradients of pathological changes in fish), which has been isolated and stocked previously in the laboratory. Alongside, two corresponding groups of fish as a control group (Table 1) were also included in this challenge test, and these naive fish were injected with 0.1 ml of sterile physiological saline (PS) (0.85% NaCl) following the same protocol that was used to inject the bacterial treatment groups. After that, fish were monitored for 13 consecutive days after the challenge test. Mortality and morbidity were recorded daily. Alongside, any unusual behavior and clinical signs or symptoms of the fish have been monitored daily. Water temperature and pH were also recorded and monitored during the post-challenge observation.

### Sampling

After 13 days of the post-challenge period, fish from all experimental groups were euthanized using a standardized laboratory protocol to facilitate sampling. Necroscopy involved both the gross external and internal examinations prior to taking the samples of the target organs. The collected sample consisted of skin, muscle, liver, and intestine, and was preserved in 10% buffered formalin until it was processed for further histopathological examination.

### Histological observation

The samples fixed with 10% buffered formalin were first prepared for dehydration, then cleared, and subsequently infiltrated in an Automatic Tissue Processor (Shandon, Citadel 1000). After that, the samples were embedded with melted wax and sectioned using a microtome (Leica Jung Rm 2035) at a thickness of 5 µm. The sections were then placed on glass slides and set on a hot plate to dry at 37°C for 1 min. Then, staining was performed with hematoxylin and eosin, and the sections were mounted with Canada balsam as follows [24]. Finally, the photographs of the histological slides were taken using Carl Zeiss Microscopy, GmbH, Axiocam ERc 5s. The histological photographs were then carefully assessed following a blinding process to avoid bias effects in the observation. All the data and images were then processed for further comparative analysis.

## Results

**Figure 1.**
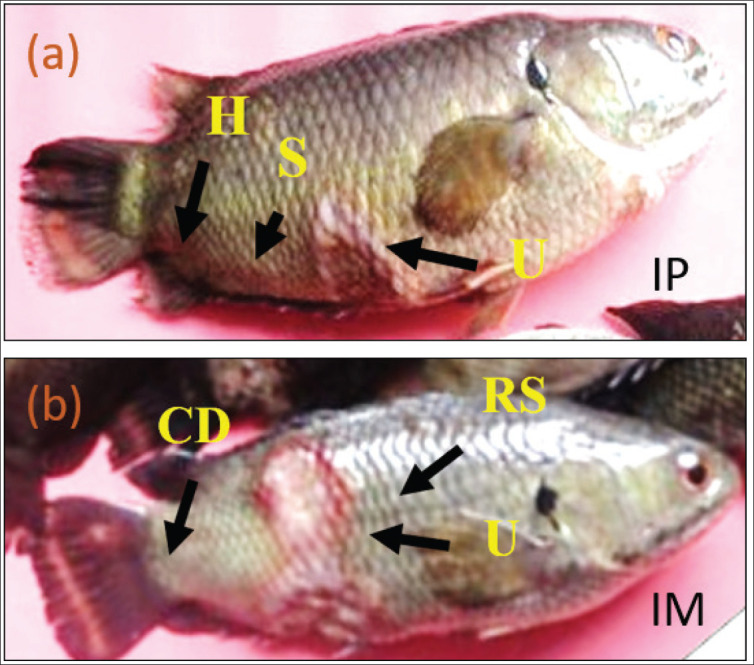
Clinical pathology observed in *A*. *testudineus* at a challenge dose of 2.1 × 106 cfu ml^−1^ from a) IP-injected and b)IM-injected fish, where H = Hemorrhage, S = Spores, U = Ulcer, CD = Caudal deformity, and RS = Rough Skin in the experimental koi fish.

Throughout the study period, the water temperature ranged from 27°C to 31°C, and the water pH ranged from 7.5 to 8.4 (Supplementary Tables S2, S3). After injection, each group of IM and IP-injected fish showed irregular movement and loss of balance. The posterior end of the body had a lesion that extended up to the caudal fin region. Anal region and the fin bases were found red. Ulcers and radish spores like clinical signs were also observed in group having IP injection challenge route at a dose of 2.1 × 104 cfu ml^−1^. In the IM fish group injected with 2.1 × 10^6^ CFU/ ml, the fish showed some identical clinical signs, including rough skin with reduced mucus, and the caudal region of a few fish was found deformed (Fig. 1a, b). In the next step, mortality was recorded, and histological observations were performed on some selected organs (i.e., liver, skin-muscle, and intestine); however, the study lacks molecular confirmation of infection following the challenge test.

### Mortality

**Figure 2.**
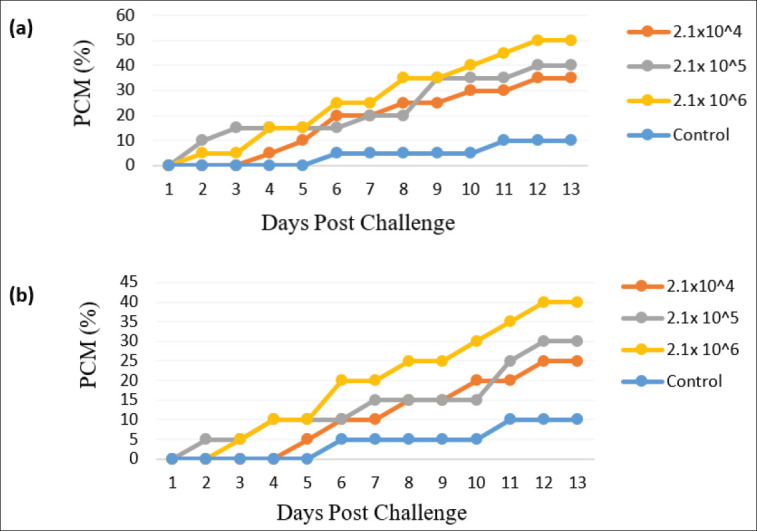
Percent cumulative mortality (PCM) of *A*. *testudineus* after 13 days post challenge with different concentrations of *A. veronii*, at a dose of 2.1 × 106, 2.1 × 106 and 2.1 × 106 cfu ml^−1^ fish following IP (a) and IM (b) injection method.

A total of 25 fish died from the IP challenge group, and a total of 19 fish died from the IM-challenged fish during the experimental period. In control, just 2 fish died (Supplementary Tables S4, S5). Cumulative mortality (CM) at bacterial concentration of 2.1 × 104, 2.1 × 106 and 2.1 × 106cfu ml^−1^ were then calculated respectively to find out the highest percent cumulative mortality (PCM %), and it was recorded in 13^th^ days of post challenge at 2.1 × 10^6^ cfu ml^−1^dose and lowest was in the control group (Fig. 2).

### Pathological changes in the liver by IP and IM methods

**Figure 3.**
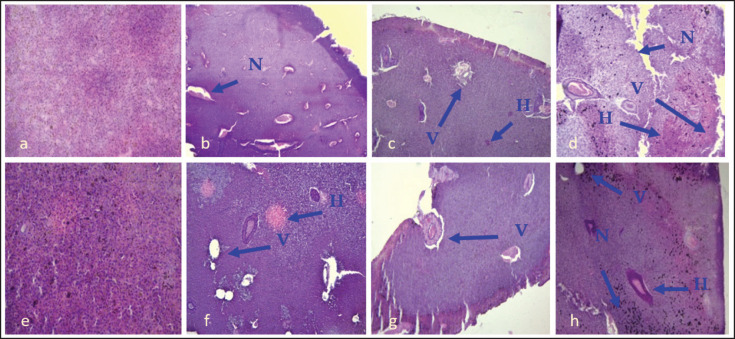
Section of liver of *A*. *testudineus* control group (H & E 120x magnification). Here, (a) and (e) are the sections of normal liver in control group, Figure (b) and (f) are demonstrating the challenge dose of 2.1 × 105 cfu ml^−1^ from IP and IM group respectively, the histopathology observations include vacuums (V), necrosis (N) and hemorrhage (H) indicated with arrows. Section c and g) represent the dose of 2.1 × 105 cfu ml^-1^ injected group having V, N and H, and section (d) and (h) are showing the dose of 2.1 × 106 cfu ml^−1^ having V, N and H in IP and IM injected fish, respectively.

In the case of IP injection, the section of the liver was almost normal in the control group (Fig. 3a, e). In both IP and IM injected group, necrosis (N), vacuums (V), and hemorrhage (H) were found in 2.1 × 104and 2.1 × 105cfu ml^−1^ injected fish (Fig. 3b, c, f, g). In case of 2.1 × 106cfu ml^−1^ injected group (both IP and IM), more pathological changes were observed (Fig. 3d, h).

### Pathological changes in skin-muscle from IP and IM challenge groups

**Figure 4.**
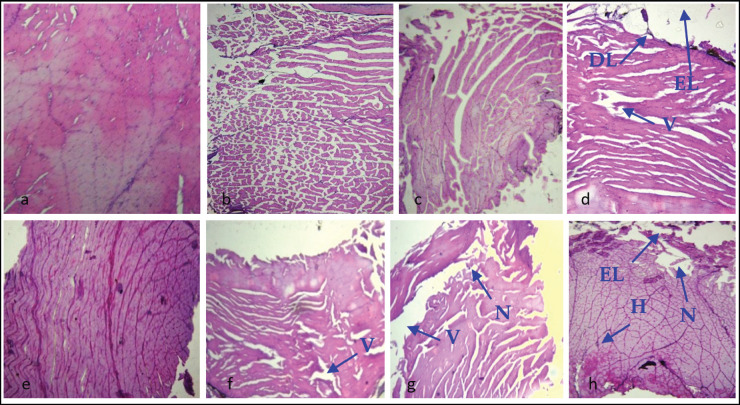
Sections of skin-muscle of A. testudineus (H & E 120x magnification). Section (a) and (e) show the skin-muscle of fish from control group having normal structure; Sections (b) to (d) belongs to IP-challenged fish and (f–h) belongs to IM-challenged fish. Here, respective arrows indicate loss of epidermis and dermis (EL, DL) and necrosis (N), vacuums (V), and hemorrhage (H) in skin-muscle

The skin-muscle interface had a normal structure in the control group (Fig. 4a, e). In 2.1 × 105cfu ml^−1^ IP and IM groups, fish skin-muscle had loss of epidermis and dermis (EL, DL) and necrosis (N) (Fig. 4b, f). In 2.1 × 105 cfu ml^−1^ injection groups (both IP and IM), vacuums (V), and necrosis (N) were present (Fig. 4c, g). In 2.1 × 106 cfu ml^−1^ challenge group, both IP and IM injected fish showed the loss of epidermis and dermis, necrosis (N), and vacums (V) in the observed histological slides (Fig. 4d, h).

### Pathological changes in the intestine by the IP and IM methods

**Figure 5.**
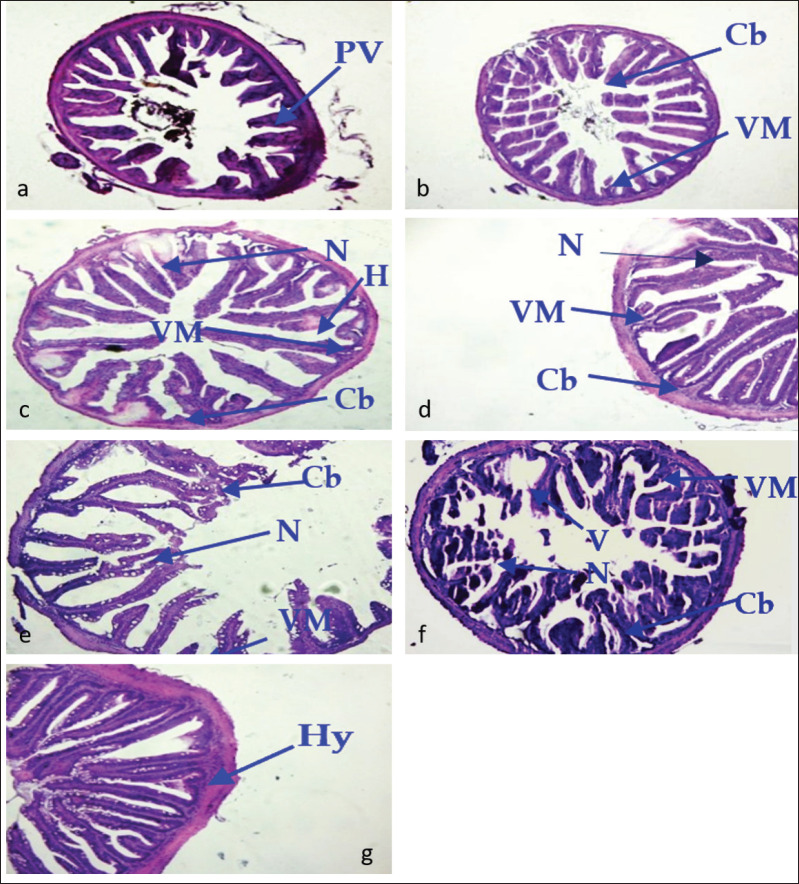
Sections of intestine of *A*. *testudineus* (H & E 120x magnification). Section (g) was from the control group, and sections (a), (b), and (c) were from IP-injected fish and sections (d), (e), and (f) show the IM-injected fish groups’ intestine. Here, the arrows are directed to pathological signs, minor hypertrophy (Hy), partial loss of villi (PV) partial missing of villi (VM), clubbed (Cb) villi and necrosis (N), and hemorrhage (H).

In the control group, histological sections of the intestine were almost normal (Fig. 5g) with minor hypertrophy (Hy). In case of 2.1 × 104 cfu ml^−1^ challenge dose, IP injection caused the partial loss of villi (PV) (Fig. 5a) and IM injected group was revealed with a partial missing of villi (VM), clubbed (Cb) villi, and necrosis (N) (Fig. 5d). On the other hand, in case of 2.1 × 105 cfu ml^−1^ injection group, partial loss of villi and clubbing were recorded in IP injected fish (Fig. 5b), and the partial loss of villi, necrosis, and clubbing were recorded in IM injected fish (Fig. 5e). In 2.1 × 10^−6^ cfu ml^−1^injection group, VM, clubbed (Cb) villi, hemorrhage (H) and necrosis (N) were documented from both IP (Fig. 5c) and IM (Fig. 5f) challenged fish in this study.

## Discussion

This experimental study was set to explore the pathogenic influences of *A*. *veronii* in climbing perch, *A*. *testudineus* through the comparison of histological observations in some specific organs such as skin-muscle, liver, and intestine under different routes of infection, consisting IP and IM with different doses- 2.1 × 106, 2.1 × 106,and 2.1 × 106 cfu ml^−1^ using experimental infection method.

Before experimental infection, the fish used in these experiments appeared to be bright and healthy. After injection*,* the challenged fish groups exhibited abnormal appearances and behavior. Clinically, external signs in fish can be evident as restlessness and erratic movement, which may be caused by stress [25]. In this study, the clinical signs observed in the infected fish included red coloration in the anal region and fin bases, reduced skin mucus, and skin lesions in the high-dose (2 × 106 cfu ml^−1^) bacterial challenge groups. Infected fish with pale body coloration and fin loss were recorded in another aquaculture species, stinging catfish *Heteropneustes fossilis*, in a study by [26]; however, due to EUS infection.

In an experimental pathogenesis test of *A*. *hydrophila* in shing, as the major clinical sign, hemorrhagic lesions were observed at the injection site, a hyperemic anal region, and hyperemic fin bases. Grayish-white lesions were detected on the caudal area of the experimental fish [27]. In another study, hemorrhagic lesions and reddish spots were observed in naturally infected exotic carp, *Barbodes* *gonionotus* [28]. This type of comparative histological study, related to experimental infection, can be used to inform further observations by providing insights into the pattern of disease signs and transmission routes in aquaculture candidates.

In this study, the highest number of dead fishes was recorded in IP-injected fish from 2.1 × 106 challenge group, and the highest percent cumulative mortality (PCM%) was recorded from 2.1 × 106cfu ml^−1^ dose in the IP-injected fish group, and the lowest was in the control group. The highest mortality in the IP-injected fish could be a result of the pathogenic effect of *A*. *veronii* on fish. However, with the increase in bacterial concentrations, mortality can also be increased, which might be the case in the present study.

The present investigation was done to examine the effect of various dilutions of *A*.* veronii* on the histopathological alterations in the liver, skin-muscle, and intestine of *A*. *testudineus*. At the end of the certain post challenge period (13 days), pathological signs were observed in the fish organs from 2.1 × 10^−^5cfu ml^−1^ injection groups (IP and IM) compared to the other bacterial suspension (2.1 × 10^−^5 cfu ml^−1^ and 2.1 × 10^−^5cfu ml^−1^). The control group had almost the normal structure in all the organs. For histological observation of skin-muscle, 2.1 × 10^−^5cfu ml^−1^ injection showed vacuum in IM group, and 2.1 × 10^−^5 cfu ml^−1^ group were observed with vacuum and necrosis in IM injected fish as features of histopathology. In skin-muscle, IP and IM injected group had partial loss of epidermis and dermis lost, necrosis, and vacuums in 2.1 × 106 cfu ml^−1^ bacterial suspension. Skin hemorrhages and necrosis were also observed in *Cyprinus carpio* after *A. hydrophila* infection [29]. The possible explanation could be *that Aeromonas sp.* produces toxins and extracellular products (e.g., hemolysin, protease, and elastase), which can cause severe necrosis and vacuolation sometimes in the liver and skin-muscle [30]. Necrotic lesions of the skin and muscle, along with inflammation and hemorrhage in walking catfish infected with *A. hydrophila* through the IM challenge test, have also been observed by [31]. The findings from the present study will have direct or practical implications regarding understanding the pathogenic nature of *A. veronii*. Thus, it can also be useful to identify critical control points for disease incidence and overall aquaculture health management, and most importantly, for disease prevention.

The liver of the control fish was almost normal, and no pathological changes were observed. But with the increased dilutions of *A*. *veronii*, pathologies in organs were increased, like hemorrhage, necrosis, and vacums. The 2.1 × 106 cfu ml^−1^ injected group revealed more prominent pathological changes (hemorrhage, necrosis and vacuums) than the 2.1 × 106 and 2.1 × 106 injected group. Liver of *A*. *testudineus* at 2.1 × 106 cfu ml^−1^ injection groups had some cells with vacuums and severe necrosis.

At the cellular level, another observation related to chronic infections caused by *A. hydrophila* has also been reported by [32], including dermal histological changes such as the presence of inflammatory cells, tissue necrosis, and cell degeneration in Nile tilapia, *Oreochromis niloticus*. In addition, authors [30] found various hemorrhages in the interstitial tissues of visceral organs and some skin hemorrhages on the ventral surface of the body and anal regions.

Mildly affected liver with hemorrhages, necrosis, and vacuoles of *A. testudineus* have also been observed by [31]. The most severe histopathological damage caused by Aeromonas sp. was also observed in the functional epithelium of the liver and kidneys, followed by the intestines [29]. In addition, they observed various hemorrhages, partial loss of villi, and necrosis, as we recorded in the interstitial tissues in the present study. The results revealed that *Aeromonas* sp.may have negative effects on the intestinal tissues of fish, which can further cause a reduction in nutrient absorption and can ultimately retards the growth and normal physiology of fish [24]. However, the present study had some limitations, including a small sample size, a lack of molecular confirmation for virulence gene detection, and the absence of quantitative scoring for the observed lesions.

## Conclusion

*Aeromonas* *veronii* is increasingly gaining importance as a serious emerging pathogen for the aquaculture and ornamental fish industries. This research was conducted to observe the effects of *A*. *veronii* on clinical and histological changes of the organs of *A*.* testudineus*. The main findings of this study revealed that freshwater fish are the most susceptible to the pathogenic effects of *Aeromonas*, as several *Aeromonas* species are commonly found in diverse freshwater environments. Our results clearly demonstrate that characteristic histopathological variations in the liver, muscle, and intestine can serve as a reliable biomarker for evaluating *Aeromonas* bacterial disease in farmed *A*.* testudineus*. We recommend routine histopathological screening in cultured *A*. *testudineus* populations for early detection of *Aeromonas* infections and further research into mitigation strategies towards comprehensive aquaculture health management and timely policy interventions.

## Figures and Tables

**Supplementary Table S2:** Record of water temperature °C at various doses of injection during the experimental period.

Dose \ Day	Injected bacterial suspension													
	2.1 × 104 (IP)	2.1 × 104 (IM)	2.1 × 104 (IM)	2.1 × 105 (IM)	2.1 × 105 (IP)	2.1 × 105 (IP)	2.1 × 105 (IM)	2.1 × 105 (IM)	2.1 × 106 (IP)	2.1 × 106 (IP)	2.1 × 106 (IM)	2.1 × 106 (IM)	Control1	Control2
1	30	30	30	30	29	30	30	29	30	29	30	30	29	30
2	28	28	28	28	27	27	28	28	28	28	28	28	27	28
3	29	29	29	29	29	29	29	29	29	29	29	28	28	28
4	31	31	31	31	31	31	31	31	31	31	31	31	31	30
5	27	27	27	27	27	27	27	27	27	27	27	28	27	28
6	28	28	28	28	28	29	28	28	28	28	28	28	27	27
7	30	30	30	29	30	30	30	30	30	30	29	30	29	30
8	29	29	29	29	28	29	29	29	30	29	29	28	28	27
9	28	29	28	28	29	29	28	28	27	28	28	28	28	28
10	27	28	27	27	27	27	28	27	27	27	27	27	26	27
11	27	27	28	29	28	28	28	29	28	28	27	28	28	29
12	31	30	31	31	30	32	31	30	31	31	31	31	31	31
13	30	30	30	29	29	29	30	29	29	29	30	29	29	29

**Supplementary Table S3:** pH observation.

Dose\Day	Injected bacterial suspension													
	2.1 × 104 (IP)	2.1 × 104 (IP)	2.1 × 104 (IM)	2.1 × 104 (IM)	2.1 × 105 (IP)	2.1 × 105 (IP)	2.1 × 105 (IM)	2.1 × 105 (IM)	2.1 × 106 (IP)	2.1 × 106 (IP)	2.1 × 106 (IM)	2.1 × 106 (IM)	Control1	Control2
1	8.4	8.0	8.1	8.3	8.4	8.2	7.9	8.0	7.9	8.1	8.2	8.1	8.3	8.1
2	8.2	7.9	7.8	7.8	7.5	8.1	8.0	8.0	7.9	8.1	8.2	8.0	8.3	8.1
3	8.1	8.1	7.9	8.1	7.7	8.3	7.9	8.0	8.0	8.3	8.0	7.8	8.4	8.0
4	8.4	8.2	8.1	8.2	7.6	8.4	8.0	8.3	8.0	8.3	8.2	8.0	8.4	8.0
5	8.0	8.0	8.2	8.3	7.6	8.2	8.1	8.4	8.0	8.3	8.1	8.0	8.4	8.1
6	8.4	8.2	8.3	8.1	7.8	8.1	8.1	8.3	8.1	8.4	8.2	8.0	8.4	8.2
7	8.3	8.1	8.2	7.4	8.1	7.9	8.1	8.1	8.1	8.4	8.2	8.1	8.2	8.2
8	8.4	8.0	8.4	8.1	8.2	8.0	8.2	8.1	8.1	8.4	8.1	8.1	8.2	8.1
9	8.0	8.1	8.0	8.2	8.0	8.1	8.2	8.1	8.3	8.4	8.2	8.1	8.4	8.0
10	7.8	84	7.5	8.3	8.4	8.3	8.2	8.1	8.3	8.3	8.1	8.2	8.2	8.2
11	8.4	8.3	8.1	8.0	8.1	8.1	8.1	8.2	8.3	8.3	8.2	8.2	8.2	8.2
12	8.1	8.5	8.2	8.0	8.3	8.4	8.1	8.2	8.2	8.3	8.1	8.1	8.4	8.3
13	8.0	8.1	7.6	8.4	8.4	8.4	8.1	8.3	8.2	8.3	8.2	8.1	8.4	8.4

**Supplementary Table S4:** Mortality of fish at various doses of IP and IM injections during the study period.

Days \ Post Challenge	Mortality (NDF = Number of dead fish)						
	Control	IP	IM	IP	IM	IP	IM
2.1 × × 104	2.1 × × 106	2.1 × × 105	2.1 × × 105	2.1 × × 106	2.1 × × 106
1	0	0	0	0	0	0	0
2	0	0	0	2	1	1	0
3	0	0	0	1	0	0	1
4	0	1	0	0	1	2	1
5	0	1	1	0	0	0	0
6	1	2	1	0	0	2	2
7	0	0	0	1	1	0	0
8	0	1	1	0	0	2	1
9	0	0	0	3	0	0	0
10	0	1	1	0	0	1	1
11	1	0	0	0	2	1	1
12	0	1	1	1	1	1	1
13	0	0	0	0	0	0	0
Total	2	7	5	8	6	10	8

**Supplementary Table S5:** PCM (%) from the experimental challenge and control groups.

Days post challenge	IP_2.1 × 104 PCM (%)	IP_2.1 × 105 PCM (%)	IP_2.1 × 106 PCM (%)	IM_2.1 × 104 PCM (%)	IM_2.1 × 105 PCM (%)	IM_2.1 × 106 PCM (%)	Control PCM (%)
1	0	0	0	0	0	0	0
2	0	10	5	0	5	0	0
3	0	15	5	0	5	5	0
4	5	15	15	0	10	10	0
5	10	15	15	5	10	10	0
6	20	15	25	10	10	20	5
7	20	20	25	10	15	20	5
8	25	20	35	15	15	25	5
9	25	35	35	15	15	25	5
10	30	35	40	20	15	30	5
11	30	35	45	20	25	35	10
12	35	40	50	25	30	40	10
13	35	40	50	25	30	40	10

## List of abbreviations

%, percentage; µl drop⁻¹, microliter per drop; °C, degree Celsius; Cb, clubbed villi; CD, caudal deformity; cfu ml⁻¹, colony-forming unit per milliliter; CM, cumulative mortality; DL, loss of dermis; EL, loss of epidermis; EUS, epizootic ulcerative syndrome.; gm, gram; H&E 120x, hematoxylin and eosin stain at 120x magnification; H, hemorrhage; h, hours; Hy, hypertrophy; IM, intramuscular; IP, intraperitoneal; L, liter; ml, milliliter; N, necrosis; NaCl, sodium chloride; PS, physiological saline; PV, partial loss of villi; RS, rough skin; S, spores; TSA, tryptic soy agar; U, ulcer; V, vacuoles; VM, missing of villi.

## Supplementary Information S1.

Bacterial preparation following the drop count method was performed as follows-

To prepare the required bacterial suspension, the cultured bacteria were diluted 10-fold seven times with distilled water. Then, the replicate drops (20 µl drop^−1^) from each dilution were placed onto TSA plate, which was previously divided into 6 portions. Then, the plates were kept until it dry, and finally, the plates were placed in the incubation unit at 25°C for at least 24 h. After the incubation period, visible colonies were observed carefully, and the count was performed; the average number of colonies per drop (cfu ml^−1^)was revealed using the following formula:

cfu ml^−1^ = N_c__−_ X D_f_ × 50; Where, N_c_ = Average number of colonies and D_f_ =Dilution factor.
